# Novel Methicillin-Resistant *Staphylococcus aureus* CC8 Clone Identified in a Hospital Setting in Armenia

**DOI:** 10.3389/fmicb.2019.01592

**Published:** 2019-07-10

**Authors:** Dorota Jamrozy, Raju Misra, Zhen Xu, Mary M. Ter-Stepanyan, Karine S. Kocharyan, Rory Cave, Armen Dz Hambardzumyan, Hermine V. Mkrtchyan

**Affiliations:** ^1^Wellcome Sanger Institute, Saffron Walden, United Kingdom; ^2^Core Research Laboratories, Molecular Biology, Natural History Museum, London, United Kingdom; ^3^Tianjin Key Laboratory of Environment, Nutrition and Public Health, Tianjin, China; ^4^Department of Toxicology and Sanitary Chemistry, Tianjin Medical University, Tianjin, China; ^5^Department of Epidemiology, Yerevan State Medical University, Yerevan, Armenia; ^6^School of Health, Sport and Bioscience, University of East London, London, United Kingdom

**Keywords:** methicillin-resistant *Staphylococcus aureus*, SCC*mec*, CC8, Papulation genetics, WGS

## Abstract

Whole-genome sequencing (WGS) of methicillin-resistant *Staphylococcus aureus* (MRSA) has been sparse in low- and middle-income countries, therefore, its population structure is unknown for many regions. We conducted a pilot surveillance of MRSA in the maternity ward of a teaching hospital in Armenia, to characterize the genotypes of circulating MRSA clones. In total, 10 MRSA isolates from a hospital environment (*n* = 4) and patients (*n* = 6) were recovered between March and May 2015 and April and May 2016, respectively. WGS analysis showed that the isolates belonged to two clonal complexes (CCs): CC8 (*n* = 8) and CC30 (*n* = 2). MRSA CC30 isolates carried staphylococcal cassette chromosome *mec* (SCC*mec*) type IVa, whereas MRSA CC8 revealed a type-V_T_-related SCC*mec*, which contained a CRISPR/Cas array and showed a high similarity to SCC*mec* found in coagulase-negative staphylococci. All but one MRSA CC8 isolates carried a plasmid identical to the pSK67 and four also carried a pathogenicity island similar to SaPI5. Phylogenetic analysis showed that the MRSA CC8 isolates formed a monophyletic cluster, which emerged around 1995 and was distinct from representatives of globally-distributed MRSA CC8 lineages. WGS characterization of MRSA in countries with no previous *S. aureus* genomic surveillance can therefore reveal an unrecognized diversity of MRSA lineages.

## Introduction

*Staphylococcus aureus* is a commensal bacterium, associated primarily with nasal carriage that can be either transient or long-term (Kluytmans et al., 1997; Miller et al., 2014). It is also a human pathogen and a major cause of hospital-associated infections (Magill et al., 2014; Tong et al., 2015). The burden of *S. aureus* disease on healthcare systems is commonly attributed to antibiotic-resistant isolates, in particular methicillin-resistant *S. aureus* (MRSA) (De Kraker et al., 2011). Transmission of MRSA in a healthcare setting can occur directly from a colonized patient or indirectly through environmental contamination or a colonized healthcare worker (Coll et al., 2017).

Global emergence of methicillin-resistant clones has been a major driver of the recent *S. aureus* evolution. Methicillin resistance is most often mediated by carriage of the *mecA* gene that encodes a penicillin binding protein PBP2a and is located on a mobile genetic element (MGE) known as staphylococcal cassette chromosome *mec* (SCC*mec*) (Katayama et al., 2000). Currently, there are 13 SCC*mec* types recognized, identified based on combination of the *mec* and *ccr* gene complexes (IWG-SCC, 2009; Wu et al., 2015; Baig et al., 2018). Recent evidence indicates that SCC*mec* emerged in coagulase-negative staphylococci (CoNS), more specifically in the *S. sciuri* group (Rolo et al., 2017). CoNS harbor high diversity of SCC*mec* elements and may act as a reservoir of SCC*mec* for *S. aureus* (Zong et al., 2011; Tulinski et al., 2012). Although multiple, independent acquisitions of various SCC*mec* types by distinct genetic backgrounds of *S. aureus* have occurred, the global population structure of MRSA is highly clonal and dominated by a few highly successful lineages (Monecke et al., 2011). Pandemic lineages of *S. aureus* include clonal complex (CC) 5 and CC8 and multiple MRSA strains emerged from both genomic background (Monecke et al., 2011).

Whole-genome sequencing (WGS) has been instrumental in studying the population structure of MRSA (Harris et al., 2010). However, while WGS of *S. aureus* has been widely implemented in some countries, for many regions its population genetics are largely unknown. Armenia is a low–middle income country and currently no MRSA infection control program exists in this country and no formal population surveillance is being carried out in patients entering the hospitals with signs or symptoms of illness compatible with *S. aureus* disease (Mkrtchyan et al., 2017). Previously, we reported the occurrence of diverse MRSA genotypes in Armenia, represented by both pandemic and sporadic lineages including CC8, CC30, CC45, and CC182 (Mkrtchyan et al., 2017). In this study, we provide further insights into the genomic background and phylogenetic origins of MRSA isolates in Armenia based on WGS analysis.

## Materials and Methods

### Study Protocol

Samples were collected during two pilot surveillance studies, conducted to identify and characterize genotypes of MRSA isolates circulating in a hospital setting in Armenia. The studies were conducted in the maternity ward of a 130-bed teaching hospital. Environmental samples (*n* = 390) were collected between March and May 2015, from baby scales, patient examination chairs, telephones, antibacterial lamps, surgical tables, nurse laboratory coats, and taps. Patient samples (*n* = 135, single sample per patient) were collected between April and May 2016. Samples were taken from patients within 48 h of hospital admission, from feces, throat, skin lesions, or wounds. A single clinically significant isolate per sample was characterized. The research protocol and informed consent was approved by the Ethics Committee of the Yerevan State Medical University after Mkhitar Heratsi (approval YSMU 24.04.2016/No. 6). All research was performed in accordance with the relevant guidelines and regulations. Informed consent was obtained from all participants and/or their legal guardians.

### Isolate Identification and Susceptibility Testing

Isolates were initially selected using mannitol salt agar (MSA; Oxoid Ltd., Basingstoke, United Kingdom) and a latex agglutination test (ProLab Diagnostics, Neston, United Kingdom). Putative staphylococci isolates were confirmed as *S. aureus* using a matrix-assisted laser desorption ionization time flight mass-spectroscopy (MALDI-TOF-MS) as described previously (Mkrtchyan et al., 2013). Isolates were tested by a disk diffusion method for susceptibility to penicillin (1 unit), cefoxitin (10 mg), and oxacillin (1 mg), according to the Clinical Laboratory Standard Institute (CLSI) protocol (Clinical and Laboratory Standard Institute [CLSI], 2013). MRSA was defined as *S. aureus* that showed resistance to cefoxitin.

### Whole-Genome Sequencing, Genome Assembly, and Annotation

Genomic DNA was extracted using the MasterPure complete kit (Epicentre, Madison, WI, United States) according to the manufacturer’s instructions, eluted in 50 μl 0.1× TE buffer (Sigma, St. Louis, MO, United States) and stored at −80°C.

Libraries were prepared using the Nextera XT library preparation kit (Illumina, San Diego, CA, United States) according to the manufacturer’s instructions. Sequencing was performed on Illumina MiSeq platform. The short-read data were deposited in the European Nucleotide Archive (ENA), under the study PRJEB19432. Individual sample accession numbers are provided in Table 1.

**TABLE 1:** Summary of MRSA isolates characterized in this study.

**Accession**	**ST**	**CC**	**SCC*mec***	**Sample date**	**Source**
ERR2750861	8	8	V	May 2016	Throat swab
ERR2750862	8	8	V	May 2016	Urine
ERR2750863	30	30	IVa	May 2016	Feces
ERR2750864	8	8	V	May 2016	Skin lesion
ERR2750866	30	30	IVa	May 2016	Feces
ERR2750867	8	8	V	May 2016	Wound
ERR1837613	8	8	V	May 2015	Baby scale
ERR1837616	8	8	V	May 2015	Table
ERR1837617	8	8	V	May 2015	Nurse’s lab coat
ERR1837618	8	8	V	May 2015	Tap

The raw FASTQ data were quality trimmed using Trimmomatic v0.35 (Bolger et al., 2014). Trimmed FASTQ reads were assembled using SPAdes v3.7.1 (Bankevich et al., 2012) and the assembled contigs were annotated with Prokka v1.11 (Seemann, 2014), using the genus *Staphylococcus* for the annotation reference.

### Genotyping and Accessory Genome Analysis

Multi-locus sequence typing and analysis of antimicrobial resistance gene carriage was performed by sort-read mapping with SRST2 (Inouye et al., 2014).

Sequences representing SCC*mec* regions were identified based on presence of the *mecA* gene and manually extracted from annotated assemblies.

Additional accessory genomic regions representing putative MGEs were further identified from whole-genome assemblies by whole-genome alignment against CC-specific reference genomes: MRSA252 (Holden et al., 2004) representing CC30 and USA300_FPR3757 (Diep et al., 2006) representing CC8. Alignment was performed using blastn 2.7.0+ (Camacho et al., 2009). For each assembly, contigs with a query coverage per unique subject (qcovus) value below an arbitrary cut-off of 95% were investigated further. Such contigs were found to represent either chromosomal fragments, containing a region of difference between a reference and a queried assembly, or complete/truncated MGE sequences. A database of unique, putative MGE sequences was curated manually. Sequence identity between a putative MGE sequence and publicly deposited genome data was checked using the NCBI BLAST and the GenBank nucleotide sequence database (NCBI Resource Coordinators, 2016). Distribution of identified MGEs among isolates was analyzed with SRST2 (Inouye et al., 2014).

To identify carriage of MGEs that might be shared between isolates and a reference genome, isolates were mapped with SRST2 against a reference-specific MGE sequence database.

### Maximum-Likelihood Inference of CC8 and CC30 Phylogenies

To reconstruct maximum-likelihood (ML) phylogenetic trees of *S. aureus* CC30 and CC8, sequence reads were mapped to CC-specific reference genomes, MRSA252 and USA300_FPR3757, respectively, with SMALT v0.7.4 (SMALT, 2017). For each CC, a core genome alignment was created after excluding MGE regions, variable sites associated with recombination [detected with Gubbins (Croucher et al., 2014)], and sites with >5% of uncalled variants. A phylogenetic tree was generated with RAxML v8.2.8 (Stamatakis, 2014) based on generalized time reversible (GTR) model with GAMMA method of correction for among site rate variation.

### Bayesian Inference of CC8 Phylogeny

To reconstruct a time-calibrated phylogeny of *S. aureus* CC8, a core genome alignment was created as described for the ML tree, and the phylogenetic analysis was performed with BEAST v2.4.7 (Bouckaert et al., 2014). The tips were dated with the year of isolation. The analysis was performed under a GTR+Γ nucleotide substitution model, with a strict clock and a coalescent Bayesian skyline model. The MCMC chain was run for 50 million generations, sampling every 1000 states. Log and tree files from five independent runs were combined using LogCombiner and analyzed with Tracer v1.5. A maximum clade credibility (MCC) tree was generated with TreeAnnotator.

### Inclusion of *S. aureus* Genome Data From Previous Studies

For the phylogenetic analysis, whole-genome sequence data of previously published *S. aureus* isolates was included. For the ML phylogeny of *S. aureus* CC8 and CC30, all isolates from the EARSS study (Aanensen et al., 2016) that represented the same CC were included (*n* = 28 and *n* = 35, respectively). For the time-calibrated phylogeny of *S. aureus* CC8, to create a geographically and temporally diverse snapshot of *S. aureus* CC8 population, isolates were selected from three published studies: the EARSS report (Aanensen et al., 2016), a study on the global origin of community-acquired *S. aureus* ST8 (Strauß et al., 2017), and a study describing the CC8 population genetics in the United States (Jamrozy et al., 2016) as well as four reference genomes, each representing a different CC8 sub-lineage: USA300_2014.C01 (GenBank accession: CP012119), 2395 USA500 (Benson et al., 2014), M1 (Larner-Svensson et al., 2013), and VC40 (Sass et al., 2012; Supplementary Table S1).

## Results

### Characterization of MRSA Isolates

In total, 47 *S. aureus* isolates were recovered during two pilot surveillance studies, including 18 from environmental sources in 2015 and 29 from patients in 2016. Ten (21%, four in 2015 and six in 2016) were identified as MRSA and characterized by WGS. MLST analysis revealed two CCs: CC8 (all ST8; *n* = 8) and CC30 (all ST30 *n* = 2) (Table 1). MRSA CC8 isolates were identified among environmental samples from 2015 and clinical samples from 2016 (Table 1). In contrast, the two MRSA CC30 were isolated only from patient feces in 2016. All MRSA isolates carried the *blaZ* and *mecA* beta-lactam resistance genes. No other antimicrobial resistance genes were identified in the analyzed isolates.

### Characterization of SCC*mec* Elements in MRSA CC8 and CC30 Isolates

Methicillin-resistant *S. aureus* CC30 isolates carried SCC*mec* type IVa, for which a full sequence was extracted from an assembly of a representative isolate. The element was 24 kb in size, and shared a 99% sequence identity and 97% coverage with the SCC*mec* IVa region of the ACME-SCC*mec* composite island from the USA300 strain R92 (Sabat et al., 2013).

**FIGURE 1:**
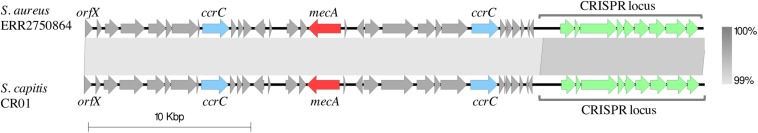
Comparison of the SCC*mec*-CRISPR structures from a representative of MRSA CC8 clone isolated in Armenia (ERR2750864) and the *S. capitis* strain CR01 (GenBank accession: LN866849.1). The sequence of the 37 kb SCC*mec*-CRISPR region from the Armenian MRSA CC8 isolate was aligned against the SCC*mec*-CRISPR fragment from the *S. capitis* strain CR01 (coordinates:413424 – 451712). Arrows represent open-reading frames. The following selected genes are annotated: the chromosomal *orfX* (the 3′-end of this gene represents SCC*mec* insertion site), *ccrC* encoding the cassette chromosome recombinase C, *mecA* encoding the penicillin-binding protein PBP2a that mediates the methicillin resistance and the CRISPR region. Gray blocks between elements indicate regions of shared sequence similarity, shaded to represent the % identity (range: 99–100%). The alignment figure was generated with Easyfig (Sullivan et al., 2011).

All MRSA CC8 isolates carried a 38-kb type V_T_-related SCC*mec*, containing two *ccrC* genes (Boyle-Vavra et al., 2005). It also contained a clustered regularly interspaced short palindromic repeats (CRISPRs) element in the J1 region. A complete sequence for this element was extracted from an assembly of a representative isolate. The entire SCC*mec*/CRISPR sequence was highly similar (99% sequence identity and 100% coverage) to a previously described SCC*mec* found in the *Staphylococcus capitis* strain CR01 from a case of neonatal sepsis in France (Figure 1; Simões et al., 2013). It also resembled a CRISPR-carrying SCC*mec* type V found in the *Staphylococcus schleiferi* strain TSCC54 that was isolated from a dog skin in Japan (99% sequence identity and 98% coverage) (Sasaki et al., 2015). Additionally, a 27-kb fragment of the SCC*mec* region containing the SCC*mec* type V_T_ was highly similar (99% sequence identity and 99% coverage) to SCC*mec* type V_T_ from *Staphylococcus pseudintermedius* strain 06-3228 (Black et al., 2009), isolated from a dog skin in the United States.

### Characterization of Other MGEs in MRSA CC8 and CC30 Isolates

All MRSA isolates identified in this study were checked for presence of additional accessory regions and MGEs other than SCC*mec*. Whole-genome assemblies were compared against CC-specific reference genomes to screen for carriage of MGEs found in the reference genomes as well as to identify unique contigs. MRSA CC30 sequences were compared against the MRSA252 genome (Holden et al., 2004). This revealed sharing of SaPI4, a pathogenicity island-like element, the *blaZ*-carrying Tn*552* transposon and an integrated plasmid, which contains heavy metal and cadmium resistance genes, *arsBC* and *cadAC*. Unique genome regions found in MRSA CC30 isolates, based on alignment against the MRSA252 genome, represented mostly phage sequences and did not reveal any other virulence or antimicrobial resistance-associated MGEs.

Methicillin-resistant *S. aureus* CC8 isolates were compared against the USA300_FPR3757 reference genome (Diep et al., 2006) but no MGE sharing was observed except for two isolates that carried a phiSa3 type phage with high similarity to phiSa3USA. All MRSA CC8 isolates from Armenia were negative for the *pvl* genes encoding Panton–Valentine leukocidin. Analysis of genome sequences not shared between MRSA CC8 isolates and the reference genome revealed presence of several putative MGEs, some carrying virulence-associated and metal resistance genes. All but one of the MRSA CC8 isolates carried a 27-kb plasmid. A contig representing complete plasmid sequence was identified in a representative isolate and was used for subsequent homology searches. The plasmid sequence showed a high similarity to the pSK67 plasmid (99% sequence identity and 100% coverage), that was first identified in a *S. aureus* isolate collected in 1949 in Australia (Gillespie et al., 1985). Identical plasmids were also reported more recently such as the pSK67-M1, found in *S. aureus* strain M1 from Denmark (Larner-Svensson et al., 2013) and the 19321-p03, identified in a USA300 isolate from the United States (Kennedy et al., 2010). This 27-kb plasmid contains a penicillin resistance *blaZRI* operon, cadmium resistance genes *cadCD*, as well as a virulence-associated cluster composed of three staphylococcal enterotoxin genes: *seD*, *seJ*, and *seR*. Four of the MRSA CC8 isolates also carried the enterotoxin genes *seK* and *seQ* located on a staphylococcal pathogenicity island (SaPI). SaPI sequence was identified within a chromosomal contig as a region of difference between the reference and isolate genome. The identified fragment was 10 kb in size and it was truncated at one end. However, terminal region containing the integrase gene was intact and the insertion site was located downstream of an ABC-transporter operon (locus ID in USA300_FPR3757: SAUSA300_0796 to SAUSA300_0798). This corresponds to the insertion site of SaPI5 found in the USA300_FPR3757 reference genome, although the two SaPI elements shared low sequence similarity (98% sequence identity and 39% coverage). Instead, SaPI fragment from Armenian MRSA CC8 was more closely related to a pathogenicity island from the USA500 reference genome (Benson et al., 2014) (95% sequence identity and 94% coverage). The remaining four *seKQ*-negative as well a single *seKQ*-positive MRSA CC8 isolate carried a phage with enterotoxin gene *seA.* In addition, all MRSA CC8 isolates carried a SaPI-like element inserted downstream of a rpsR gene (locus ID in USA300_FPR3757: SAUSA300_0368) encoding a ribosomal protein S18, although it did not contain any known virulence genes.

### Phylogenetic Analysis of MRSA CC8 and CC30 Isolates

To investigate whether the identified MRSA isolates from the same CC are closely related, short reads were mapped to a CC-specific reference genome to determine the pairwise single-nucleotide polymorphism (SNP) distance. In addition, phylogenetic relationship between the isolates from the same CC was reconstructed and analyzed in the context of a CC-specific population structure. For this, we combined MRSA genomes from Armenia with previously published dataset from the European Antimicrobial Resistance Surveillance System (EARSS) report on invasive *S. aureus* in Europe (Aanensen et al., 2016).

**FIGURE 2:**
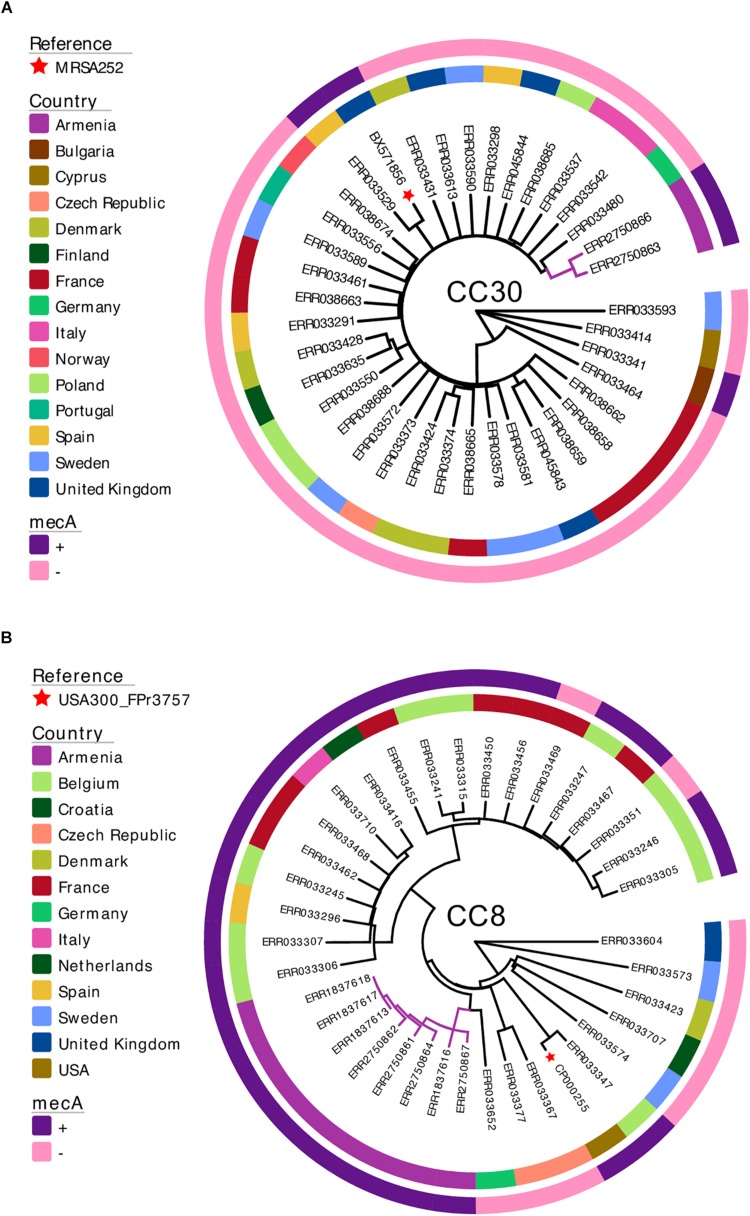
Maximum-likelihood phylogenetic trees of *S. aureus* isolates from Armenia and other countries. The two MRSA CC30 **(A)** and eight MRSA CC8 **(B)** isolates from Armenia were combined with genomes of *S. aureus* isolates collected and sequenced as part of a previously published European Antimicrobial Resistance Surveillance System study. Each phylogenetic tree is annotated with a color strip representing the country of origin (inner ring) and presence/absence of the *mecA* gene (outer ring). Tips representing reference genomes are annotated with a star. The tree figure was generated with Evolview (Zhang et al., 2012; He et al., 2016).

The two MRSA CC30 isolates identified in this study were non-clonal, with a pairwise SNP distance of 96. Phylogenetic analysis using the external dataset (*n* = 35) showed that the isolates from Armenia were most closely related to a *mecA*-negative isolate from Germany (Figure 2A). Pairwise SNP distance between this German CC30 isolate and the two isolates from Armenia was 255 and 268.

The MRSA CC8 isolates from Armenia revealed a closer phylogenetic relationship. The median pairwise SNP distance between the eight isolates was 70. Three isolates were clonal showing no genetic variation and all were collected in 2015 from environmental sources. Phylogenetic tree of MRSA CC8 together with the external dataset representing *S. aureus* CC8 population from Europe (*n* = 28) showed that all isolates from Armenia formed a single monophyletic cluster (Figure 2B), and were most closely related to a *mecA*-negative *S. aureus* from Germany. Median pairwise SNP distance between this isolate and the Armenian MRSA cluster was 253.

### Evolutionary History of MRSA CC8 Isolates

**FIGURE 3:**
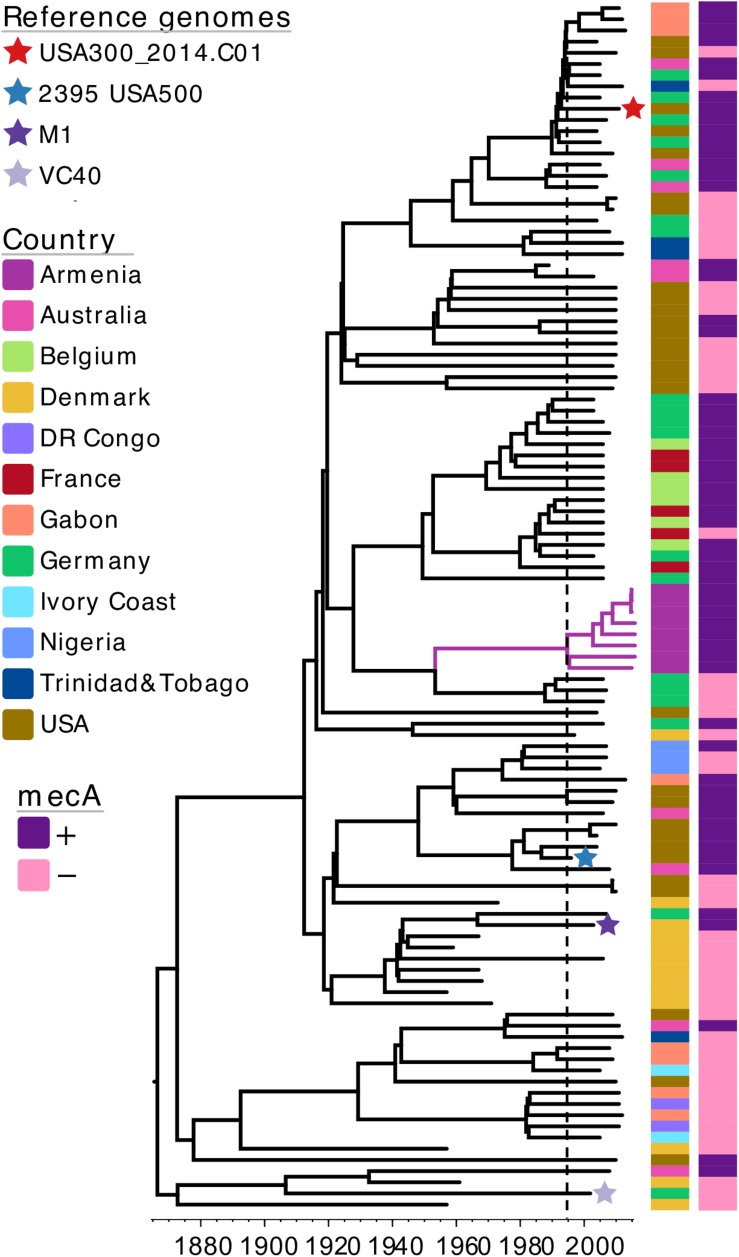
A maximum clade credibility tree of *S. aureus* CC8 isolates from Armenia and other countries. The tree is annotated with color strips representing isolate country of origin and presence/absence of the *mecA* gene. Tips representing reference genomes included in the analysis are annotated with a star. Vertical dashed line indicates the estimated time-point for the emergence of Armenian MRSA CC8 cluster. The tree figure was generated with Evolview (Zhang et al., 2012; He et al., 2016).

As majority of MRSA isolates identified in this study belonged to CC8, with all isolates representing a single, unique phylogenetic cluster, we wanted to investigate more closely the evolutionary origins of this MRSA CC8 sub-lineage. For this, we again combined our dataset with previously published *S. aureus* genomes, expanding the external dataset to create a more diverse snapshot of CC8 population. In addition to isolates from the EARSS study, we also included selected genomes from a study on the global origin of community-acquired *S. aureus* ST8 (Strauß et al., 2017), data from a study describing the CC8 population genetics in the United States (Jamrozy et al., 2016) as well as four reference genomes, each representing a different CC8 sub-lineage (Supplementary Table S1). As such, the MRSA CC8 from Armenia was combined with a total of 100 previously published *S. aureus* CC8 genomes (Supplementary Table S1). To estimate the time of the most recent common ancestor (TMRCA) of the Armenian MRSA CC8 cluster we reconstructed a time-calibrated phylogeny (Figure 3). The core genome alignment contained 11,329 SNPs and the estimated mutation rate was 1.06 × 10^–6^ substitutions per site per year [95% confidence interval (CI), 9.69 × 10^–7^–1.15 × 10^–6^), which is in agreement with the previously reported molecular clock estimates for CC8 (Uhlemann et al., 2014). The TMRCA for the Armenian cluster was around 1995 (95% CI, 1992–1999), indicating that this MRSA sub-lineage emerged recently. It was phylogenetically distinct from the MRSA USA300 and USA500 clones, represented on the tree by the USA300_2014.C01 and the 2395 USA500 reference genomes, respectively. Instead, the Armenian CC8 sub-lineage was more closely related to a MRSA clade represented by isolates from France, Germany, and Belgium although the TMRCA for the two clusters was around 1928 (95% CI, 1920–1935). Based on this CC8 phylogeny, reconstructed using a more geographically diverse collection of *S. aureus* isolates, the Armenian cluster was still most closely related to MSSA isolates from Germany, with the TMRCA for the two clusters estimated around 1953 (95% CI, 1946–1960).

## Discussion

We report a WGS-based characterization of MRSA isolated from samples that were collected from patients and the environment in a teaching hospital in Armenia during two pilot surveillance studies in 2015 and 2016. Isolates represented two *S. aureus* lineages, CC8 and CC30. Only two MRSA CC30 isolates were identified, both collected in 2016 from fecal samples indicating association with asymptomatic carriage rather than the hospital setting. In contrast, the MRSA CC8 isolates were identified from both environmental and clinical samples that were collected at the two time-points, suggesting a persistence of this clone within the surveyed hospital. This MRSA CC8 clone was phylogenetically distinct from other MRSA CC8 clusters, derived from external datasets, indicating an independent emergence from a MSSA population. While the Armenian clone was most closely related to a cluster of MSSA isolates from Europe, the distant TMRCA between the two clusters suggests that the Armenian MRSA more likely emerged from a distinct MSSA population. Additional sampling of *S. aureus* isolates in Armenia would be required to determine whether this MRSA CC8 clone emerged from a local MSSA background.

The clone carried a type-V_T_-related SCC*mec*, which showed the highest similarity to SCC*mec* from two reference genomes of CoNS, derived from diverse sources and geographic locations. This inter-species carriage suggests that the element has a high capacity for horizontal dissemination. It might also confer a specific selective advantage as the *S. capitis* strain CR01, carrying this SCC*mec*, represents the NRCS-A clone that has been linked with clonal spread in neonatal intensive care units (Butin et al., 2016). This particular SCC*mec* variant might therefore play an important role in mediating a transmission within a hospital setting.

The MRSA CC8 isolates from Armenia revealed additional genomic features that can confer further selective advantage and enhance their pathogenicity. Furthermore, similar or identical elements have been previously observed in other MRSA CC8 clones. Plasmid with high sequence similarity to pSK67, carried by all but one Armenian MRSA CC8 isolate, was also identified in a representative of a USA300 clone from the United States (Kennedy et al., 2010). It was also found in the *S. aureus* M1 strain defined as the t024-ST8-IVa clone associated with MRSA outbreak in Denmark (Larner-Svensson et al., 2013). In addition, the *seKQ*-carrying pathogenicity island, that was observed in four of the Armenian MRSA CC8 isolates, shared a high sequence similarity to SaPI5 from the USA500 clone. Although the Armenian MRSA CC8 was phylogenetically distinct, it showed a similar pattern of accessory genome adaptation associated with other MRSA CC8 clones.

## Conclusion

The main limitation of this study is a small sample size of the characterized MRSA isolates. Despite this, it provides preliminary insights into the genetic structure of MRSA circulating in a hospital setting in Armenia. Additional surveillance studies are being conducted to further our understanding of genetic diversity of MRSA in the healthcare and community settings in Armenia and to identify possible risk factors, antimicrobial resistance prevalence, and the burden of MRSA disease in this country. Further genomic surveillance of MRSA isolates is also needed to understand better the epidemiology of the described here MRSA CC8 clone in Armenia.

## Data Availability

The short-read data were deposited in the ENA, under the study PRJEB19432 (https://www.ebi.ac.uk/ena/data/view/PRJEB19432).

## Ethics Statement

The research protocol and informed consent was approved by the Ethics Committee of the Yerevan State Medical University after Mkhitar Heratsi (approval YSMU 24.04.2016/No. 6).

## Author Contributions

HM: conceptualization and design of the study, data analysis, writing, and critically reviewing the manuscript. DJ: WGS data analysis, and writing and critically reviewing the manuscript. RM: whole-genome sequencing, data analysis, and reviewing the manuscript. ZX, RC, and AH: laboratory work and reviewing the manuscript. MT-S: sample collection, laboratory work, and reviewing the manuscript. KK: sample collection and laboratory work. All authors read and approved the final manuscript.

## Conflict of Interest Statement

The authors declare that the research was conducted in the absence of any commercial or financial relationships that could be construed as a potential conflict of interest.
